# Predictors of malignancy in patients with solitary pulmonary nodules undergoing pulmonary resection

**DOI:** 10.1111/crj.13489

**Published:** 2022-04-26

**Authors:** Eren Erdogdu, Berker Ozkan, Salih Duman, Melek Agkoc, Sukru Mehmet Erturk, Murat Kara, Alper Toker

**Affiliations:** ^1^ Department of Thoracic Surgery Istanbul University Faculty of Medicine Istanbul Turkey; ^2^ Department of Radiology Istanbul University Faculty of Medicine Istanbul Turkey; ^3^ Department of Cardiovascular and Thoracic Surgery West Virginia University Heart and Vascular Institute Morgantown West Virginia USA

**Keywords:** lung carcinoma, malignancy, predictor, solitary pulmonary nodule

## Abstract

**Background:**

The management of a solitary pulmonary nodule is a challenging issue in pulmonary disease. Although many factors have been defined as predictors for malignancy in solitary pulmonary nodules, the accurate diagnosis can only be established with the permanent histological diagnosis.

**Objective:**

We tried to clarify the possible predictors of malignancy in solitary pulmonary nodules in patients who had definitive histological diagnosis.

**Methods:**

We made a retrospective study to collect the data of patients with solitary pulmonary nodules who had histological diagnosis either before or after surgery. We made a statistical analysis of both the clinic and radiological features of these nodules with respect to malignancy both in contingency tables and with logistic regression analysis.

**Results:**

We had a total of 223 patients with a radiological diagnosis of solitary pulmonary nodule. Age, smoking status and pack years of smoking, maximum standardized uptake value (SUVmax), and radiological features such as solid component, spiculation, pleural tag, lobulation, calcification, and higher density were significant predictors of malignancy in contingency tables. Age, smoking status and smoking (pack/year), SUVmax, and radiological features including spiculation, pleural tag, lobulation, calcification, and higher density were the significant predictors in univariate analysis. However, multivariate analysis revealed only SUVmax greater than 2.5 (*p* < 0.0001), spiculation (*p* = 0.009), and age older than 61 years (*p* = 0.015) as the significant predictors for malignancy.

**Conclusion:**

Age, SUVmax, and spiculation are the independent predictors of malignancy in patients with solitary pulmonary nodules.

## 1 INTRODUCTION

Solitary pulmonary nodules (SPNs) are increasingly found in daily practice with the growing number of high‐resolution imaging modalities particularly computerized tomography (CT) of the chest. The prediction of the nature of an SPN is of paramount importance for an appropriate approach. Optimal management of these nodules might provide an early diagnosis and appropriate treatment for patients with malignant tumors, which might also minimize unnecessary interventions and procedures for those with benign nature.

An SPN is a round intraparenchymal lung lesion that is less than 3 cm in diameter, which is surrounded by normal lung tissue without showing any sign of atelectasis and lymphadenopathy. Chest X‐rays may reveal an SPN in 1 out of 500 examinations, and almost 90% of these SPNs were found incidentally.^1^ Differential diagnosis of an SPN ranges from primary lung cancer or metastases of extrathoracic malignancies to infections and other benign lesions.^2^ Most of these SPNs are benign lesions accounting for a rate of 50% to 70%; however, the remaining nodules are malignant, which show a potential to invade and spread through the lymphatics of the lung and further metastasize to distant organs. Thus, the prediction of probability of malignancy for an SPN is of utmost significance, which is closely related to early diagnosis and treatment for a possible lung cancer.

We conducted a retrospective study to outline the potential predictors of malignancy in patients with an SPN who underwent surgery. We statistically analyzed the clinical and radiological features of these SPNs to clarify the significant predictors of malignancy.

## 2 MATERIALS AND METHODS

### 2.1 Clinical features

We have a total 223 patients with a radiologically proven SPN during a period from April 2005 to October 2019 in the Department of Thoracic Surgery, in Istanbul University Faculty of Medicine. Patients are 139 (62.3%) males and 84 (37.7%) females with a mean age of 58.2 ± 11.2, and a median age of 61 (range, 15 to 82) years (Table S1). Among these patients, 179 (80.3%) are smokers. They have a history of smoking with a mean of 38.7 ± 24, and a median of 35 pack/year. A total of 124 (55.6%) patients have SPN, which are located at the upper lobes. The most common localization of SPN is the right lung in 127 (57%) cases. We excluded the cases with mediastinal lymph node enlargement, a history of malignancy in the recent 5 years, and a radiological sign of atelectasis. None of the patients showed ^18^F‐fluorodeoxyglucose‐positron emission tomography (FDG‐PET) positive mediastinal lymph nodes. A total of nine (4%) patients showed pathologically proven N1 disease, whereas only one patient had N2 disease in the postoperative period.

### 2.2 Radiological features

Two thoracic surgeons and a radiologist have independently assessed the images of CT. The radiological mean of SPN size is 20.3 ± 6.3 (range, 8 to 30) mm. The mean density of solid nodules was 46.6 ± 26.1 (range, −17 to 177). The mean of maximum standardized uptake value (SUVmax) is 7.1 ± 6 (range, 0 to 46). The nodules were also evaluated according to their radiological features in thorax CT. The presence of solid component, spiculation, pleural tag, lobulation, calcification, cavitation, and density based on Hounsfield unit (HU) scale were recorded. Solid component was categorized as solid and subsolid nodules. Subsolid nodules included both pure ground‐glass opacity (GGO) and partly solid GGO lesions.

### 2.3 Diagnostic procedures and histopathological features

A total of 152 (68.2%) patients were referred either or both to pulmonology and interventional radiology departments for preoperative diagnostic procedures such as fiberoptic bronchoscopy (FOB) or a transthoracic fine needle biopsy (TFNB). Histologic diagnosis was obtained with a TFNB and a bronchoscopic biopsy in 75 (33.6%) and 12 (5.3%) of these cases, respectively. Preoperative histological diagnosis with TFNB was an adenocarcinoma, squamous cell carcinoma, non‐small cell carcinoma, malignant tumor, and a hamartoma in 36 (48%), 19 (25.3%), 16 (21.3%), 3 (4%), and 1 (1.3%), respectively. Bronchoscopic biopsy showed a squamous cell carcinoma and a carcinoid tumor in 3 (25%) and 9 (75%) cases, respectively.

Preoperative diagnosis was an indeterminate SPN without any definitive histologic diagnosis in 136 (61%) patients. Among these patients, those with malignant diagnosis at frozen section obtained by a wedge resection underwent an anatomic lung resection.

Video‐assisted thoracoscopic surgery (VATS) was the most common procedure in 178 (79.8%) patients, whereas an open thoracotomy was the choice of procedure in 45 (20.2%) patients. We performed a lobectomy in 149 (66.8%) patients, segmentectomy in 31 (14%) patients, wedge resection in 27 (12.1%) patients, bilobectomy in 5 (2.2%) patients, enucleation in 5 (2.2%) patients, and sleeve lobectomy in 6 (2.7%) patients. Among patients who underwent sleeve lobectomy, three had N1 positivity with pericapsullary invasion of the lymph nodes, and two had tumor positivity at the bronchial resection margin. The remaining patient underwent a bronchial stump repair who showed obstruction of the middle lobe bronchus following stapling of the right lower lobe bronchus. We performed systematic mediastinal lymph node dissection provided that the patient has been proven to have malignant nodule either at preoperative work‐up or frozen section examinations. We dissected mediastinal stations of 2R, 4R, 7, 8, 9, 10R for right‐sided tumors, and 5, 6, 7, 8, 9, 10L for left‐sided tumors.

On histological examination, a total of 185 (83.0%) cases showed a malignant pulmonary nodule. The most common definitive malignant diagnosis was adenocarcinoma in 109 (58.9%) patients followed by squamous cell carcinoma in 51 (27.6%) and carcinoid tumor in 17 (9.2%) patients. Hamartoma was the most common benign diagnosis in 24 (63.6%) patients (Table S1). The mean of histopathological tumor size was 21.8 ± 8 (range, 4 to 36) mm. Pathological staging was stage IA in 135 (73%) patients, stage IB in 29 (15.7%) patients, stage IIA in 3 (1.6%) patients, stage IIB in 15 (8.6%) patients, and stage III in 2 (1.1%) patients among the malignant cases.

### 2.4 Statistical analysis

Age, gender, smoking status, radiological diameter, SUVmax, localization, and radiological features were included in the assessment of predictors of malignancy. Age, smoking status, tumor size, SUVmax, and density were classified as a high or low group relative to the median value.

The Kolmogorov–Smirnov test was used to determine the distribution of the continuous data. Categorical variables were analyzed with the Chi‐square and Fisher's exact tests as appropriate in contingency tables, whereas Student's *t*‐test or Mann–Whitney *U*‐test was performed as appropriate for comparison of continuous variables. The stepwise logistic regression analysis was applied for univariate and multivariate analysis to confirm the impact of the clinical and radiological factors on malignancy. Using backward selection, we achieved a final reduced model by eliminating variables that were not statistically significant at a level of 0.05. Data were expressed as the mean ± standard deviation. A *p* value less than 0.05 was considered statistically significant. All statistical analyses were performed with the Statistical Package for Social Sciences (SPSS, Version 25.0, Chicago, IL, USA).

## 3 RESULTS

Age, smoking status and smoking (pack/year), and SUVmax were the significant predictors of malignancy for SPN in contingency tables (Table 1). The mean age of the cases with malignant SPN was significantly greater than that of the cases with benign SPN (*p* ≤ 0.0001). Patients aged older than 61 years (*p* ≤ 0.0001), who had smoking status of more than 30 pack/year (*p* = 0.016), and ever smokers (*p* = 0.014) had a significantly greater rate of malignant SPN. The mean SUVmax of malignant SPN was significantly greater than that of benign SPN (*p* ≤ 0.0001). In addition, subgroups of SUVmax had a significant difference regarding the malignancy. Nodules with SUVmax value greater than 2.5 appeared to have more probability of malignancy in all subgroups of increasing diameter (Table S2). On the other hand, subsolid lesions (*p* = 0.006), spiculation (*p* ≤ 0.0001), pleural tag (*p* ≤ 0.0001), and lobulation (*p* ≤ 0.0001) were significant predictors of malignancy. Calcification was a significant predictor and more commonly detected in benign (*p* = 0.016). Solid lesions with greater density had a significantly greater rate (*p* = 0.002) (Table 2).

**TABLE 1:** Clinical features of solitary pulmonary nodules with respect to malignancy

Clinicopathological features		Benign, *N* (%)	Malignant, *N* (%)	*p* value
Total		38 (17)	185 (83.0)	
Age (years)		51.1 ± 10.3	59.6 ± 10.9	**<0.0001**
Age (years)	≤61	32 (84.2)	96 (51.9)	
	>61	6 (15.8)	89 (48.1)	**<0.0001**
Gender	Male	19 (50)	120 (64.9)	
	Female	19 (50)	65 (35.1)	0.085
Smoking status	Ever	25 (65.8)	154 (83.2)	
	Never	13 (34.2)	31 (16.8)	**0.014**
Smoking status (pack/year)	≤30	28 (73.7)	97 (52.4)	
	>30	10 (26.3)	88 (47.6)	**0.016**
Smoking status (pack/year) (among smokers)	≤35	17 (68)	76 (49.4)	
	>35	8 (32)	78 (50.6)	0.083
Diameter in mm		20.1 ± 6.9	20.4 ± 6.2	0.790
Diameter in mm	≤10	3 (7.9)	13 (7)	
	10–20	19 (50)	84 (45.4)	
	>20	16 (42.1)	88 (47.6)	0.827
SUVmax value		2.8 ± 2.9	7.9 ± 6.1	**<0.0001**
SUVmax value	≤2.5	21 (65.6)	23 (12.9)	
	>2.5	11 (34.4)	155 (87.1)	**<0.0001**
SUVmax value	≤5.5	27 (84.4)	78 (43.8)	
	>5.5	5 (15.6)	100 (56.2)	**<0.0001**
Localization	Upper lobe	18 (47.4)	106 (57.3)	
	Non‐upper lobe	20 (52.6)	79 (42.7)	0.262
Localization (side)	Right	23 (60.5)	104 (56.2)	
	Left	15 (39.5)	81 (43.8)	0.625

*Note:* Bold values denote statistical significance at the *p* < 0.05 level.

Abbreviation: SUVmax, maximum standardized uptake value.

**TABLE 2:** Radiological features of solitary pulmonary nodules with respect to malignancy

Radiological features		Benign, *N* (%)	Malignant, *N* (%)	*p* value
Nature	Solid	38 (100)	156 (84.3)	
	Partly solid	‐	25 (13.5)	
	GGO	‐	4 (2.2)	**0.023**
Nature 2	Solid	38 (19.6)	156 (84.3)	
	Subsolid	‐	29 (15.7)	**0.006**
Spiculation	Spiculated	15 (39.5)	150 (81.1)	
	Non‐spiculated	23 (60.5)	23 (18.9)	**<0.0001**
Pleural tag	Absent	31 (81.6)	80 (43.2)	
	Present	7 (18.4)	105 (56.8)	**<0.0001**
Lobulation	Lobulated	18 (47.4)	143 (77.3)	
	Non‐lobulated	20 (52.6)	42 (22.7)	**<0.0001**
Calcification	Absent	21 (55.3)	138 (74.6)	
	Present	17 (44.7)	47 (25.4)	**0.016**
Cavitation	Absent	34 (89.5)	164 (88.6)	
	Present	4 (10.5)	21 (11.4)	0.573
Density		36 ± 26.6	49.2 ± 25.4	**0.005**
Density	≤45	28 (73.7)	71 (45.5)	
	>45	10 (26.3)	85 (54.5)	**0.002**

*Note*: Bold values denote statistical significance at the *p* < 0.05 level.

Abbreviation: GGO, ground‐glass opacity.

Univariate analysis revealed age (*p* = 0.001), smoking status (*p* = 0.016) and smoking (pack/year) (*p* = 0.019), subgroups of SUVmax (*p* < 0.0001), spiculation (*p* < 0.0001), pleural tag (*p* ≤ 0.0001), lobulation (*p* < 0.0001), absence of calcification (*p* = 0.018), and density (*p* = 0.003) as significant predictors of malignancy (Table 3). However, age greater than 61 years (*p* = 0.023), SUVmax greater than 2.5 (*p* < 0.0001), and spiculation (*p* = 0.012) were the only independent significant predictors for malignancy in multivariate analysis (Table 4).

**TABLE 3:** Univariate analysis of predictors by logistic regression analysis model

Variable	Relative risk	95% confidence interval	*p* value
Age (years) (>61 vs. ≤61)	4.944	1.974–12.387	***0.001***
Gender (male vs. female)	1.846	0.931–3.732	0.088
Smoking status (ever vs. never)	2.583	0.179–0.839	***0.016***
Smoking status (pack/year) (>30 vs. ≤30)	2.540	1.167–5.528	***0.019***
Tumor size (mm) (>20 vs. ≤20)	1.247	0.616–2.526	0.539
SUVmax value (>2.5 vs. ≤2.5)	12.866	5.494–30.127	***<0.0001***
SUVmax value (>5.5 vs. ≤5.5)	6.923	2.549–18.802	***<0.0001***
Localization (upper lobe vs. others)	1.491	0.740–3.003	0.264
Localization (right vs. left)	1.194	0.625–1.194	0.625
Spiculation (spiculated vs. non‐spiculated)	6.571	3.112–13.874	***<0.0001***
Pleural tag (present vs. absent)	5.812	2.435–13.877	***<0.0001***
Lobulation (lobulated vs. non‐lobulated)	3.783	1.835–7.801	***<0.0001***
Calcification (absent vs. present)	2.377	1.157–4.883	***0.018***
Density (>45 vs. ≤45)	3.352	1.525–7.370	***0.003***

*Note*: Bold values denote statistical significance at the *p* < 0.05 level.

Abbreviation: SUVmax, maximum standardized uptake value.

**TABLE 4:** Multivariate analysis of predictors by logistic regression analysis model

Variable	Relative risk	95% confidence interval	*p* value
Age (years) (>61 vs. ≤61)	5.074	1.250–20.600	**0.023**
Smoking status (ever vs. never)	1.639	0.385–6.970	0.503
Smoking status (>30 vs. ≤30 pack/year)	1.579	0.396–6.291	0.518
SUVmax value (>2.5 vs. ≤2.5)	11.285	3.729–34.148	**<0.0001**
Spiculation (spiculated vs. non‐spiculated)	5.123	1.440–18.223	**0.012**
Pleural tag (present vs. absent)	1.515	0.405–5.670	0.537
Lobulation (lobulated vs. non‐lobulated)	2.137	0.595–7.679	0.245
Calcification (absent vs. present)	2.678	0.884–8.116	0.082
Density (>45 vs. ≤45 HU)	2.918	0.936–9.096	0.065

*Note*: Bold values denote statistical significance at the *p* < 0.05 level.

Abbreviations: HU, Hounsfield unit; SUVmax, maximum standardized uptake value.

## 4 DISCUSSION

Estimation of malignant probability of an SPN has always been an important issue in pulmonary medicine because a malignant SPN means an early lung cancer with a possible early surgical removal and favorable survival outcome. Thus, the predictors of malignancy are of particular significance to make solid decisions whether follow‐up or biopsy an SPN for histologic diagnosis. For a suspicious malignant SPN, a number of methods such as TFNB, transbronchial needle aspiration biopsy, or VATS are currently available to provide histological diagnosis. However, these are all invasive and operator‐dependent procedures with varying rates of diagnostic accuracy.^2^, ^3^, ^4^ Thus, the decision for a biopsy of an SPN should be determined under very reliable clinical predictors for malignancy.

Previous data showed that age, smoking history, and tumor history are highly indicative of high malignant risk factors of SPN.^5^, ^6^ Similarly, we found that age older than 61 years as an independent predictor of malignancy in our study. In addition, radiologic imaging is usually necessary to estimate the malignant probability of an SPN. The most common imaging modality used to assess an SPN is CT. Size and shape of the nodule have been reported as the most important factors for malignancy.^7^, ^8^ Another specific independent risk factor is the maximum diameter of the SPN. Moreover, density, margin, and calcification are other important CT features for an SPN. High‐density solid nodules have low probability of malignancy compared with GGO lesions.^8^ Nodules with lobular and irregular margins with pleural indentation are very likely to be malignant, whereas calcified nodules usually tend to be benign.^9^ Significant nodule enhancement that is equal or more than 15 HU on CT scan is a strong predictive factor of a malignant SPN.^10^ Growth rate of an SPN might also be helpful for the differential diagnosis. Doubling time of an SPN ranging from 1 month to 1 year would be highly suggestive of malignancy.^11^ Although positron emission tomography‐computerized tomography (PET‐CT) has a significant role in revealing the nature of pulmonary nodules,^12^ the estimating effect of PET‐CT for nodules less than 1 cm is still under debate. A recent research also found that plasma miRNAs provided potential circulating biomarkers for noninvasively diagnosing lung cancer in patients with SPNs.^13^ In addition, a recent report has shown that serum levels of Cyfra21‐1 were an independent risk factor for malignancy in SPN.^14^

A cost‐effective mathematical risk model is of utmost importance for chest physicians and thoracic surgeons to prevent expensive examinations and complex time‐consuming follow‐ups as well as to better determine an invasive approach in terms of survival and legal purposes. However, these models may differ from one region to another. Although upper lobe localization is a risk factor for a possible malignant SPN in some of the international risk models,^4^, ^10^ our analysis did not reveal this feature as a risk factor (*p* = 0.262) similar to Peking University People's Hospital (PKUPH) model.^9^ This finding might be attributed to the comparably more common granulomatous infections in our country, which are more commonly located in the upper lobes and resulting in false positive findings for a benign SPN.

Mayo model, Veterans Affair (VA) model, and PKUPH model are the three most frequently cited models recently.^4^, ^9^, ^10^ Among these, the Mayo model has reported a total of six independent risk factors including age, smoking history, history of extrapulmonary tumors, maximum diameter, location of the nodule, and spiculation.^10^ However, the Mayo model is limited from region and ethnicity. The Mayo model also excluded patients with previous 5‐year history of lung cancer or extrapulmonary tumors, which resulted in weakness of this study. The proportion of malignant SPN was low in this model, possibly related to the definition of malignancy at that time. Furthermore, a total of 12% of patients did not have a definite pathological diagnosis and considered as benign based on imaging findings on 2‐year follow‐ups. On the other hand, the VA model proposed age, smoking history, quitting smoking period, and diameter of the nodule as the independent risk factors.^4^ Different from other models, the VA model did not include imaging features. However, we found spiculation as a strong significant predictor of malignancy as a radiological feature in our analysis. The model of PKUPH also revealed six independent risk factors including age, maximum diameter of nodule, family history of tumor, calcification, spiculation, and tumor marginal features. This model has a more adoption to the region and ethnicity as it included the calcifications of SPN in the analysis compared with the above‐mentioned models. Nodules with calcification are usually benign; however, malignant SPN may contain eccentric calcification. The PKUPH model has been shown to have comparably high accuracy and has been recommended as the most appropriate model for SPN.^9^, ^14^

PET with ^18^F‐fluorodeoxyglucose (FDG) is very useful in the differentiation of benign and malignant SPN. It has been suggested that PET might aid to reduce the number of patients who undergo unnecessary surgical biopsy for SPN.^15^ PET using ^18^F‐FDG is an accurate and noninvasive method for differentiating benign SPN from lung cancer with a sensitivity of 95% and a specificity of 82%.^20^ Furthermore, overall diagnostic accuracy improves when CT and PET are combined as a hybrid imaging with the high sensitivity of CT and the high specificity of PET.^16^ Likewise, it has been suggested that definitive histologic diagnosis is mandatory in cases even with micronodules that are highly suggestive of malignancy following hybrid imaging.^17^ Metabolic parameters obtained from PET studies have shown to improve the prediction of malignancy in SPN.^16^, ^18^ However, a significant number of patients still undergo surgical resection to make a definitive histological diagnosis.^2^ The most significant predictor for malignancy in our multivariate analysis was a SUVmax greater than 2.5. Similar to our study, López et al. showed that SUVmax and age were the independent variables to predict malignancy.^19^

## 5 CONCLUSIONS

The management of an SPN necessitates a multidisciplinary teamwork. Although several clinical and radiological features have been proposed for the estimation of malignancy for an SPN, SUVmax appears as the most significant predictor.

## CONFLICT OF INTEREST

The authors declare no potential conflicts of interest with respect to the research, authorship, and/or publication of this article.

## ETHICS STATEMENT

Our study has been performed in accordance with the ethical standards as laid down in the 1964 Declaration of Helsinki and its later amendments or comparable ethical standards.

This retrospective study was approved by the Istanbul Medical Faculty Ethics Committee of Istanbul University by the number 1541/2019.

Written informed consent was obtained from all patients for their anonymized information to be stored in the hospital database and used for clinical research as well as to be published in this article.

## AUTHOR CONTRIBUTIONS

E.E. and M.K. collected and analyzed the data. M.K., B.O., and M.A. wrote the manuscript. E.E., B.O., and S.D. planned the study. M.A. helped collect the data, E.E., A.T., and S.M.E. analyzed the radiological features, and M.A. and M.K. revised the manuscript. All authors read and approved the manuscript.

## Supporting information


**Table S1.** Clinicopathological features of patients with solitary pulmonary nodules
**Table S2.** Nodule diameter subgroups and SUVmax with respect to malignancy

## Data Availability

The data that support the findings of this study are available on request from the corresponding author. The data are not publicly available due to privacy or ethical restrictions.
