# Supplementary material for: Predictors of malignancy in patients with solitary pulmonary nodules undergoing pulmonary resection
Source: Clin Respir J. 2022 Apr 26;16(5):361–8. doi: 10.1111/crj.13489 (PMC9366584; doi:10.1111/crj.13489)
Supplement: Supplementary file 1 — Table S1. Clinicopathological features of patients with solitary pulmonary nodules Table S2. Nodule diameter subgroups and SUVmax with respect to malignancy [file CRJ-16-361-s001.docx]

**Table S1.** Clinicopathological features of patients with solitary pulmonary nodules

|  |  | **N (%)** | **Mean ± SD** |
| --- | --- | --- | --- |
| **Gender** | Female  Male | 139 (62.3)  84 (37.7) |  |
| **Age (years)** |  |  | 58.2 ± 11.2 |
| **Smoking Status**  **(pack / year)** | Ever  Never | 179 (80.3)  44 (19.7) | 38.7 ± 24 |
| **Radiological Size (mm)** |  |  | 20.3 ± 6.3 |
| **Density (HU)** |  |  | 46.6 ± 26.1 |
| **SUVmax** |  |  | 7.1 ± 6 |
| **TFNB** ^§^ | Total  Diagnostic  Nondiagnostic | 107 (47.9)  75 (70.1)  32 (29.9) |  |
| **Histological diagnosis by TFNB**^§^ | Adenocarcinoma  Squamous cell carcinoma  Non-small cell carcinoma  Malignant tumor  Hamartoma | 36 (48)  19 (25.3)  16 (21.3)  3 (4)  1 (1.3) |  |
| **FOB** ^†^ | Total  Diagnostic  Nondiagnostic | 95 (42.6)  12 (12.6)  83 (87.4) |  |
| **Histological diagnosis by FOB** ^†^ | Carcinoid tumor  Squamous cell carcinoma | 9 (75 %)  3 (25 %) |  |
| **Surgical Procedure** | VATS  Thoracotomy | 178 (79.8)  45 (20.2) |  |
| **Type of resection** | Lobectomy  Segmentectomy  Wedge resection  Sleeve lobectomy  Bilobectomy  Enucleation | 149 (66.8 %)  31 (14 %)  27 (12.1 %)  6 (2.7 %)  5 (2.2 %)  5 (2.2 %) |  |
| **TNM Stage** | IA  IB  IIA  IIB  IIIA | 135 (73)  29 (15.7)  3 (1.6)  16 (8.6)  2 (1.1) |  |
| **N Stage** | N0  N1  N2 | 175 (94.6)  9 (4.9)  1 (0.5) |  |
| **Definitive diagnosis (Malignant)** | Adenocarcinoma  Squamous cell carcinoma  Carcinoid tumor  Large cell carcinoma  Small cell carcinoma  Sarcomatoid carcinoma  Adenosquamous cell carcinoma  Lymphoepithelioma-like carcinoma | 109 (58.9)  51 (27.6)  17 (9.2)  4 (2.2)  1 (0.5)  1 (0.5)  1 (0.5)  1 (0.5) |  |
| **Definitive diagnosis (Benign)** | Hamartoma  Tuberculosis  Necrosis  Pneumonia  Leiomyoma  Pneumocytoma  Vasculitis | 24 (63.6)  6 (15.8)  3 (7.9)  2 (5.3)  1 (2.6)  1 (2.6)  1 (2.6) |  |

^§^TFNB; transthoracic fine needle biopsy, ^†^FOB; fiberoptic bronchoscopy

Table S2: Nodule diameter subgroups and SUVmax with respect to malignancy

|  | **Malignant** | **Benign** |
| --- | --- | --- |
|  | n (%) | n (%) |
| **PET-CT (Nodules ≤ 10 mm)** | 13 | 3 |
| SUVmax ≤ 2.5 | 5 (38,5) | 2 (66,7) |
| SUVmax > 2.5 | 7 (53,8) | 0 (0) |
| PET-CT not available | 1 (7,7) | 1 (33,3) |
| **PET-CT (Nodules 10-20 mm)** | 84 | 19 |
| SUVmax ≤ 2.5 | 13 (15,5) | 11 (57,9) |
| SUVmax > 2.5 | 69 (82,1) | 5 (26,3) |
| PET-CT not available | 2 (2,4) | 3 (15,8) |
| **PET-CT (Nodules 30 mm)** | 88 | 16 |
| SUVmax ≤ 2.5 | 5 (5,7) | 8 (50) |
| SUVmax > 2.5 | 79 (89,8) | 6 (37,5) |
| PET-CT not available | 4 (4,5) | 2 (12,5) |
